# *Beauveria bassiana* Multifunction as an Endophyte: Growth Promotion and Biologic Control of *Trialeurodes vaporariorum*, (Westwood) (Hemiptera: Aleyrodidae) in Tomato

**DOI:** 10.3390/insects11090591

**Published:** 2020-09-02

**Authors:** Lorena Barra-Bucarei, Macarena Gerding González, Andrés France Iglesias, Gonzalo Silva Aguayo, Matías Guerra Peñalosa, Pedro Vergara Vera

**Affiliations:** 1 Instituto de Investigaciones Agropecuarias (INIA) Quilamapu, Av. Vicente Méndez 515, Chillán 3800062, Chile; afrance@inia.cl (A.F.I.); matias.guerra@inia.cl (M.G.P.); 2 Facultad de Agronomía, Universidad de Concepción, Chillán 3780000, Chile; mgerding@udec.cl (M.G.G.); gosilva@udec.cl (G.S.A.); 3 Facultad de Administración y Economía, Universidad Tecnológica Metropolitana, Santiago 1030000, Chile; pvergara@utem.cl

**Keywords:** entomopathogens, endophytes, biocontrol, *Solanum lycopersicum*, greenhouse whitefly

## Abstract

**Simple Summary:**

The tomato, *Solanum lycopersicum* L. has great importance worldwide for its nutritional characteristics and its antioxidant content. It is cultivated in different geographical areas, under field and greenhouse conditions, and it can be subjected to abiotic and abiotic factors that negatively affect production and quality. In this study, we evaluated the effect of five native endophytic strains of *Beauveria bassiana* on the reproduction of greenhouse whiteflies and the growth of tomatoes. The endophyte was inoculated in the substrate, and plants were afterward exposed to adult populations of the insect. For plant-growth promoter activity, the effect of endophytic strains on phosphate solubilization, iron siderophores production, plant height, and biomass was determined. The RGM-557 strain reduced the number of eggs and nymphs per cm^2^ on leaflet by 66 and 65%, respectively, compared with the control (water); while in comparison with the chemical insecticide the reduction was 32 and 55%, respectively. Most strains showed some degree of phosphate solubilization and siderophores production. Plants inoculated with strains RGM-557 and RGM-731 produced the greatest plant heights; RGM-731 obtained the greatest plant biomass. Endophytic *B. bassiana* provide important protection levels against whiteflies in tomato—in addition to promoting their growth.

**Abstract:**

The tomato, *Solanum lycopersicum* L. is one of the most consumed vegetables in the world; nevertheless, it is affected by biotic and abiotic factors that reduce its productivity. The whitefly is globally considered as the main pest under protected crop conditions, where biologic control using endophytic fungi emerges as a sustainable alternative. We evaluated the indirect effects of five native endophytic strains of *Beauveria bassiana* on the reproduction of greenhouse whiteflies and the growth of tomatoes. The plant growth substrate was inoculated with five strains of this endophyte and the resulting plants were then exposed to whiteflies afterwards. The effect that endophytic strains had on phosphate solubilization, iron siderophore production, plant height, and plant biomass were evaluated. The evaluated endophytes reduced the number of eggs per cm^2^ on leaflets compared to the control and behaved similarly to the commercial synthetic insecticide. Leaflets inoculated with strains RGM-557, RGM-644 and RGM-731 showed fewer nymphs than the control and those treated with insecticide. RGM-557 and RGM-731 produced the greatest plant heights; RGM-731 obtained the greatest plant biomass. Our study provides evidence that native endophytic strains of *B. bassiana* have a biocontrol effect on whiteflies and could be used to promote tomato growth.

## 1. Introduction

The tomato, *Solanum lycopersicum* L. is one of the most cultivated vegetables worldwide due to its low-fat content and excellent source of dietary fiber, minerals, vitamins and antioxidants [1]. It can be consumed fresh and/or processed into a wide variety of manufactured products [2]. Plant development can be negatively affected by biotic and abiotic factors, provoking decreased yields [3]. Among the biotic factors, several pests negatively affect tomato production; the greenhouse whitefly (GWF) *Trialeurodes vaporariorum* Westwood stands out due to its prolificacy and the important production losses it can cause in both greenhouse and field production. Moreover, the costs for its control are considerable if early measures are not taken. The damage produced consists of perforating plant tissues and sucking the sap directly from the vascular bundles—which leads to a decrease in photosynthetic activity, reduced vigor and loss of fruit quality (indirect damage) due to the presence of sooty mold [4]. Traditionally, synthetic insecticides from different groups have been used for GWF control, which has generated a selection of resistant populations [5,6]. Therefore, insecticides are not only relatively expensive, they have also decreased their effectiveness over time. Furthermore, the excessive and irrational use of insecticides has led to negative consequences for the environment, with especially negative effects on the soil [7,8]. Microbial agents such as entomopathogenic fungi, have emerged as a sustainable alternative for GWF control, some of which are available at a commercial level as a substitute for chemical insecticides [9]. Studies in both the laboratory and the field have provided evidence that the entomopathogenic fungus *Beauveria bassiana* (Bals.) Vuill. has potential to control GWF [10,11,12]. It is also worth noting that in recent years entomopathogenic fungi have received attention due to their ability to colonize the tissues of a number of plants, in an endophytic association of mutual benefit [13,14].

Many plants live in association with endophytic fungi [15], which live inside their tissues causing no apparent damage [16]. They are inherent organisms to plants, as they establish a symbiotic association of protocooperation with their host for mutual benefit [17]. Endophytes present important phylogenetic and lifestyle diversity traits such as: colonization, dissemination, specificity of plant host and location in the interior of diverse plant tissues [18]. Within this group, the entomopathogenic fungal endophytes (EFEs) have received much attention due to their agronomic importance [14,19]. Studies have demonstrated that these fungi have the ability to colonize diverse cultivated plants, including species like tomato, bean, corn and coffee [20,21,22]. These fungi are not obligate plant symbionts and can thus survive without plants [23]. Over recent years, there has been a considerable increase in research on EFEs due to the multiple benefits they provide to plants, including arthropod control, phytopathogen antagonism and plant growth promotion [13,17,18,24,25,26]. In terms of the mechanisms involved in growth promotion, EFEs have demonstrated to produce phytohormones (auxins), improve water transport, increase the availability of nutrients (solubilization of phosphate, potassium and siderophores production) as well as acting indirectly by activating biologic protection mechanisms and inducing systemic resistance to phytopathogens [18,27]. Within the auxins group, the indole-3-acetic acid is related to plant cell elongation, division and differentiation, and is important in regulating plant defense responses [28,29]. Different species of *Beauveria* can produce organic acids, such as oxalic and citric acids in the case of *B. caledonica* and formic, lactic, orotic, oxalic and citric acids in the case of *B. bassiana*; these organic acids change the pH of the medium and inorganic phosphorus is released [30]. *Beauveria bassiana* also produces siderophores, which play an important role against the cellular stress caused by iron deficiency; moreover, iron is required for fungal cell growth and metabolism [31].

Some studies have demonstrated that *B. bassiana* can become established as an endophyte in the leaves, stems and roots of sorghum and tomato through the inoculation of leaves, seeds or soil [32,33,34]. This fungus can provide the plant with a greater competitive ability, allowing for the expression of its genetic potential, expressed in higher rates of germination, more biomass accumulation and greater seed production [35]. Some of the most important mechanisms used by *B. bassiana* for insect control include pathogenicity, antagonism, systemic resistance and the tritrophic action associated with natural enemies, such as parasitoids [21,36]. Provided the demonstrated abilities of EFEs to control pests and diseases, in addition to promoting plant growth, the objective of this study was to evaluate the potential of native strains of endophytic *B. bassiana* as growth-promoters and their indirect antagonistic effect against *T. vaporariorum* in tomato.

## 2. Materials and Methods

### 2.1. Genetic Material

Tests were completed at the Instituto de Investigaciones Agropecuarias, INIA-Chile (Chillan, Chile). The tomato plants used were a Mykonos (Seminis, St. Louis, MO, USA) variety. Five strains of endophytic fungi were evaluated for growth promotion and their biocontrol effect on GWF (Table 1). These strains form part of the Chilean Microbial Genetic Resource Collection of INIA, and they were morphologically and molecularly identified and selected for their ability to endophytically colonize tomato and chili pepper tissues [34]. In the case of GWF, individuals were collected from a greenhouse tomato crop located in the town of Colín, Maule Region, Chile. Once identified according to their morphologic features [37], uniform populations were obtained from tomato plants grown under controlled conditions (26 ± 2 °C, HR = 65 ± 5%, photoperiod= 14:10). A hundred neonatal adults (24 h) were placed on tomato plants with 4 to 5 true leaves inside 50 × 50 × 50 cm cages covered with anti-aphid mesh (300 µm), according to the methodology described by Oreste et al. [12]. After 20 days, the plants showed leaves with 80–100 nymphs on average.

### 2.2. Fungal Inoculum

The fungal inoculum was prepared by cultivating each strain in Petri dishes (90 mm in diameter) with potato dextrose agar (PDA) incubated at 25 ± 2 °C in the dark for 7 days. After incubation, conidia were harvested from the dishes in a biosecurity cabinet and were then added to a sterile distilled water solution with Tween-20 at 0.01% (Difco^TM^, Detroit, MI, USA). The conidia concentration was estimated using a Neubauer chamber (BOECO, Hamburg, Germany) and was later adjusted to 1 × 10^7^ conidia mL^−1^ for the assays with GWF and 1 × 10^6^ conidia mL^−1^ for the growth promotion assays. The conidia viability was evaluated with the methodology proposed by Goettel and Inglis [38]. The suspensions were used for both the GWF biocontrol and growth promotion assays.

### 2.3. Endophyte Effect on Greenhouse Whitefly

Tomato seeds were disinfected for 1 min in 95% ethanol, then 3 min in 1.5% sodium hypochlorite and 1 min in 95% ethanol, finally the seeds were rinsed five times for 1 min each in sterile distilled water. The seeds were then dried on sterile absorbent paper for 1 h in a biosecurity cabinet. Ten µL of water were taken from the fifth rinse jar and cultured in Petri dishes with PDA plus chloramphenicol to verify the quality of the disinfection process. Afterwards, the seeds were sown in seed trays with a substrate composed of a mixture of perlite, peat, compost and vermiculite (1:1:1:0.5) which was sterilized twice in autoclave at 121 °C and 793 kPa for 1 h. The plants were placed in a growth chamber with conditions of 24 ± 2 °C, 65 ± 2% relative humidity and a photoperiod of 14 h of light and 10 h of darkness. When the plants had two true leaves they were transplanted to 300-mL pots with a sterile substrate similar to the seed trays. They were then placed in 50 × 50 × 50 cm cages covered in anti-aphid mesh (300 µm) located in greenhouses with controlled temperatures (16 ± 3 °C at night and 26 ± 3 °C during the day), a photoperiod of 14 h of light and 10 of darkness and a humidity of 65 ± 5%. Three days later, the plants were inoculated with five strains of *B. bassiana* endophytes. These endophyte strains were applied to the substrate through a 10 mL solution with a concentration of 1 × 10^7^ conidia mL^−1^; afterwards, the substrate was covered with aluminum foil. An absolute control was inoculated with the same quantity of sterile distilled water, 10 mL, in addition to 0.01% Tween-20 (Difco^TM^). A control with a commercial insecticide, whose active ingredient is fenoxycarb (INSEGAR^®^ 25 WG, Syngenta Crop Protection, Monthey AG, Monthey, Switzerland), was also established. In this case, a 0.6-mL solution of the insecticide was prepared in 1 L of water and 10 mL of this solution was applied to the foliage of each plant. The following day, the treated plants were placed in cages; each cage contained a plant with an average population of 80 to 100 neonatal nymphs of GWF. Plants were kept in the greenhouse for 45 days and were watered daily with 50 mL of sterile distilled water. Samples of five GWF adults were collected from the leaves and placed in humid chambers to determine the direct pathogenic effect of the five *B. bassiana* endophyte strains. Endophytic colonization was confirmed by taking 10 leaflets from each plant per treatment. These were disinfected and each leaflet was cut into 6 mm discs; these were placed in Petri dishes with Noble agar (Difco™) medium plus chloramphenicol and incubated in the dark at 25 ± 2 °C for 30 days using the method described by Barra-Bucarei [34]. The presence of fungus on the border of the disc was considered as positive, and the obtained result was determined to be the percentage of endophytic colonization.

A completely randomized block design with five replicates per treatment was used. The number of eggs and nymphs (instars III and IV) per cm^2^ of leaflet located in the middle part of the leaf was evaluated, considering two leaflets per leaf and two leaves in the midsection of each treated plant.

### 2.4. Analysis of Plant Growth-Promoting Attributes

A qualitative evaluation of the phosphate solubilizing activity of the five strains of *B. bassiana* was carried out. The ability of these strains to solubilize inorganic phosphorus [Ca_3_(PO_4_)] was determined using the phosphate medium from the National Institute of Botanical Research (NBRIP), which contains insoluble glucose, 10 g of Ca_3_(PO_4_)_2_; 5 g of MgCl_2_6H_2_O; 0.25 g of MgSO_4_7H_2_O; 0.2 g of KCl and 0.1 g of (NH_4_)_2_ SO_4_, as well as 5 g of TRP and 15 g of agar. The pH was adjusted to 7.0 and dissolved in one liter of sterile distilled water [39]. Six mm mycelium discs were placed in 8-cm Petri dishes with the NBRIP culture medium. These Petri dishes were then incubated for 10 days at 25 ± 2 °C in the dark. A completely randomized design with 10 replicates for each strain was used and the solubilization was determined by the phosphate solubilization index that corresponds to the ratio of the total diameter (colony + solubilization halo) and the diameter of the colony [40].

The production of iron siderophores was also evaluated. The chrome azurol S (CAS) technique was used to determine iron mobilization. Six mm mycelium discs of the five different strains were placed in 8-cm Petri dishes with the CAS culture medium. The Petri dishes were left in the incubator for 10 days in the dark at a temperature of 28 °C [41], ten replicates were carried out for each strain. The production of iron siderophores was determined according to the modified method of Andrews et al. [42], in which a change in color of the medium, from blue to orange implies a reduction of Fe+3 to Fe+2. Through the use of the software ImageJ (National Institute of Health, Bethesda, MD, US), an open-source image processing software, estimated the surface area that showed a change in coloration by the siderophores exudation. Then was calculated the percentage of the surface of the plate with siderophores exudation.

### 2.5. Growth Promotion In Vivo

Tomato seeds were disinfected in a similar manner to the methodology previously described when evaluating the endophyte effect on GWF. Afterwards, they were submerged for 4 h in a sterile distilled water solution with Tween-20 (0.01%) and a concentration of 1 × 10^6^ conidia mL^−1^ of each strain. In the case of the control, seeds the plants were submerged in a sterile distilled water solution with Tween-20 (0.01%). They were then sown in 300-mL pots with a sterile substrate composed of a mixture of perlite, peat, compost and vermiculite (1:1:1:0.5). The pots were maintained in growth chambers at 24 ± 2 °C, 65 ± 2% relative humidity and a photoperiod of 14 h of light and 10 h of darkness; they were watered daily for 30 days. A completely randomized design with five replicates for each treatment was used and measurements of the total plant height (cm). To determine plant biomass, the plants were dried for 48 h at 60 ± 2 °C separating aerial growth from roots. The assays were carried out twice.

### 2.6. Statistical Analysis

In the case of the growth promotion in vivo and the phosphate solubilization, the data were analyzed with a one-way analysis of variance (ANOVA) and the measurements were compared with the LSD-Fisher’s test (*p* < 0.05). For the greenhouse study, as the assumptions of normality and homogeneity in the number of eggs and nymph variables were not met, nonparametric statistics were used, applying the Kruskal–Wallis test [43]. The statistical program InfoStat version 2011 (Universidad Nacional de Córdoba, Córdoba, Argentina) was used for all cases [44]. All assays were conducted twice under the same conditions.

## 3. Results

### 3.1. Endophyte Effect on T. vaporariorum

**Figure 1:**
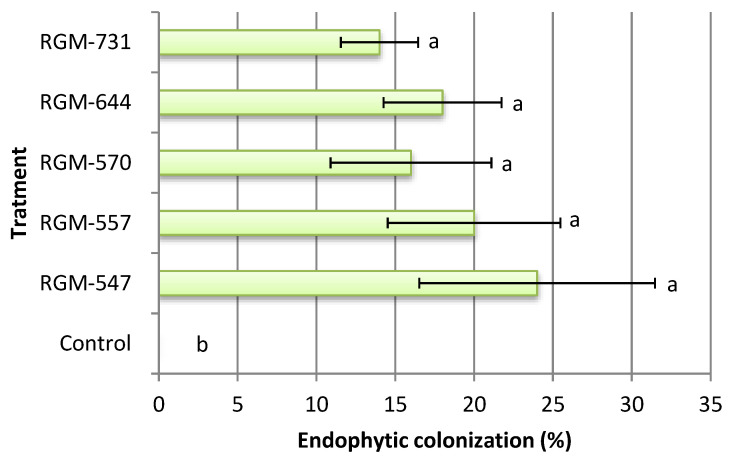
Colonization percentage of tomato by *Beauveria bassiana*, 45 days after the inoculation (*n* = 5). Bars with different letters differ according to Fisher’s LSD test (*p* < 0.05).

Significant differences were found between the plants treated with endophytes and the control plants (F = 3.1; df = 5; *p* < 0.026). The five evaluated strains internally colonized tomato leaves. White mycelium growing on leaf discs obtained from inoculated plants and placed in culture media was observed with an optical microscope and confirmed as *B. bassiana* by its morphology while in the control, the leaf discs obtained from plants showed no mycelium. The endophytic colonization fluctuated between 14% and 24%, but no significant difference was observed among the tested strains. Furthermore, 100% of the strains demonstrated a systemic mode of action, where the fungus inoculated in the roots was reisolated from the leaves (Figure 1).

**Figure 2:**
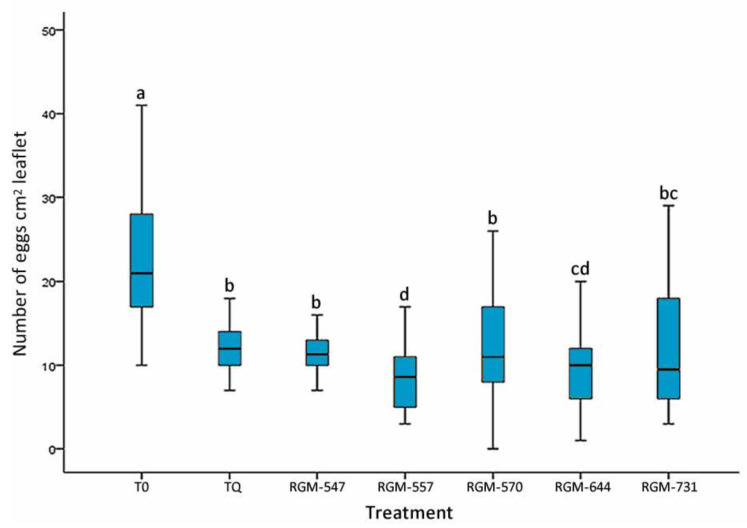
Effect of the tomato root inoculation with native strains of the *B. bassiana* endophyte on the number of eggs of *T. vaporariorum* on tomato leaves, 45 days after the inoculation (*n* = 40). T0 corresponds to control and INSEGAR^®^ 25 WG (TQ) to chemical treatment. Treatments with a common letter were not significantly different according to the Kruskal–Wallis test at *p* = 0.05. Data represent means ±SE.

Root inoculation of tomato plants with native endophyte strains of *B. bassiana* significantly reduced the number of eggs on the leaflets after 45 days of incubation in comparison to the control treated with water (T0). Pairwise comparisons (Kruskal–Wallis = 84.37, df = 40, *p* < 0.0001) showed that the plants treated with the RGM-557 & RGM-644 strains presented the least amount of eggs (*p* < 0.05), with means of 8.18 and 9.68 (N cm^2^ leaflet), respectively, when compared to plants treated with the INSEGAR^®^ 25 WG (TQ) insecticide and those treated with the RGM-547 and RGM-570 endophytes (Figure 2).

**Figure 3:**
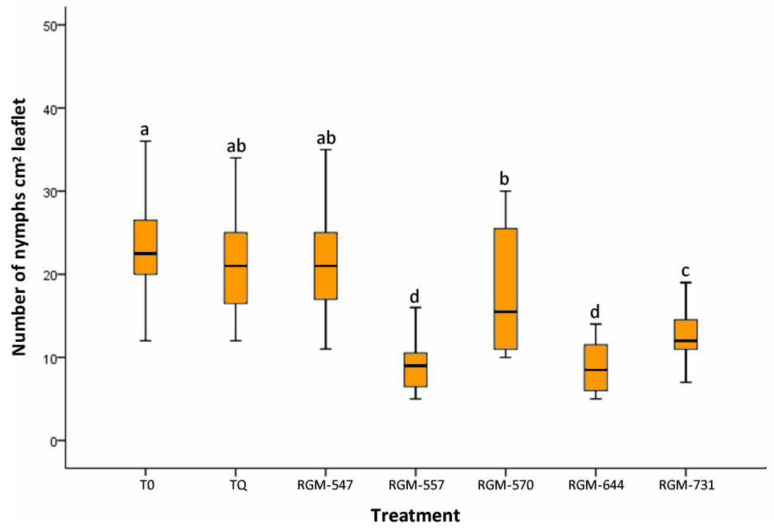
Effect of the tomato root inoculation with native strains of *B. bassiana* endophytes on the number of nymphs (instars III & IV) of *T. vaporariorum* on leaves, 45 days after the inoculation (*n* = 40). T0 corresponds to control and TQ to chemical treatment. Treatments with a common letter were not significantly different according to the Kruskal–Wallis test at *p* = 0.05. Data represent means ±SE.

In the case of the endophyte effect on the number of nymphs, a significantly lower number was also observed with respect to T0 (control) and TQ (chemical treatment) (Kruskal–Wallis = 172, df = 40, *p* < 0.0001), with the RGM-664 and RGM-557 strains again presenting the lowest number of nymphs, with means of 8.9 and 9.4 (N cm^2^ leaflet), respectively. T0 reached a means of 25.4 and TQ 21.3 nymphs per leaflet (cm^2^) (Figure 3). It is worth mentioning that the decrease in the number of nymphs observed as a result of the RGM-644 strain with respect to the TQ treatment was 58% and compared to the T0 was 65%.

### 3.2. Phosphorous Solubilization, Production of Iron Siderophores and Indole Compounds Per B. bassiana Endophyte Strain

**Figure 4:**
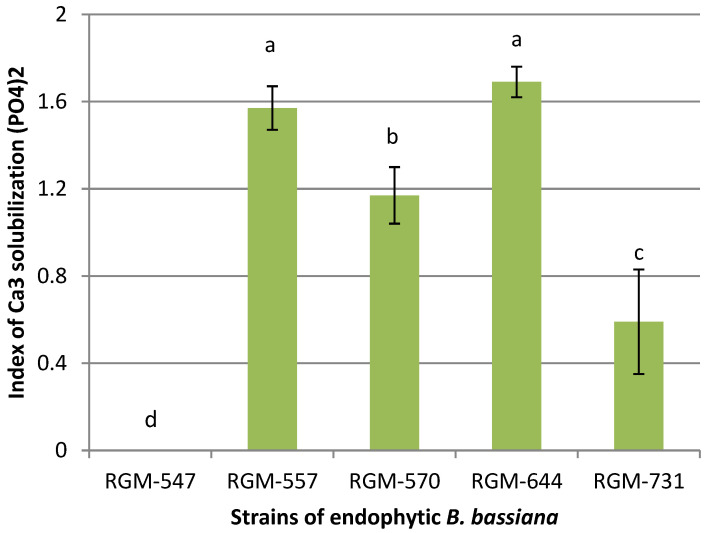
P-solubilization of Ca_3_(PO_4_)_2_ by endophytic *B. bassiana* in the agar medium National Institute of Botanical Research. Different letters over the bars represent significant differences among the treatments according to Fisher’s LSD test (*p* < 0.05).

In the in vitro studies, four strains of *B. bassiana* showed some degree of Ca_3_ solubilization (PO_4_)_2_. The phosphate solubilization index fluctuated between 0 and 1.69. The only strain that showed no solubilization was RGM-547. In the analysis of the strains that presented some degree of solubilization, the strains RGM-644 and RGM-557 presented solubilization indices of 1.57 and 1.69, significantly superior to the strains RGM-570 and RGM-731 (Figure 4).

**Figure 5:**
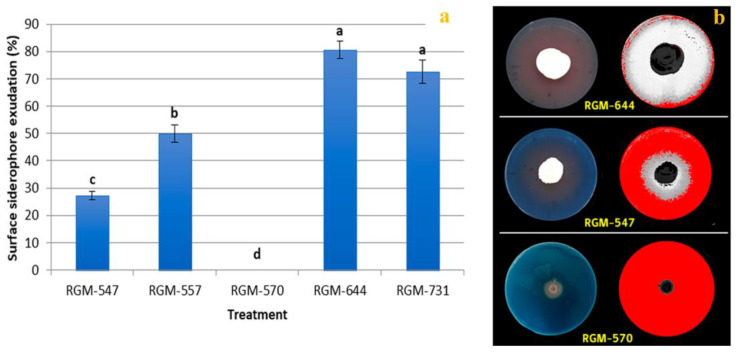
In vitro production of iron siderophores. (**a**) Surface of siderophore exudation (%) 10 days postincubation (*n* = 10). Different letters over the bars represent significant differences among the treatments according to Fisher’s LSD test (*p* < 0.05); (**b**) strain RGM-644 presented a high siderophores exudation, RGM-547 presented a low siderophores exudation and RGM-570 did not siderophores exudation, due to the absence of coloration change in medium.

For the iron siderophore production variable, the dishes inoculated with the endophytes RGM-547, RGM-557, RGM-644 and RGM-731 presented a coloration change which was considered positive by the CAS method. The dishes with the strain RGM-570 showed no coloration changes and were therefore considered negative. The endophytic *B. bassiana* strains RGM-644 and RGM-731 produced the greatest surface of siderophores exudation with 81% and 73%, respectively. (F = 55.8; df = 10; *p* < 0.001) (Figure 5).

### 3.3. Growth Promotion In Vivo

**Figure 6:**
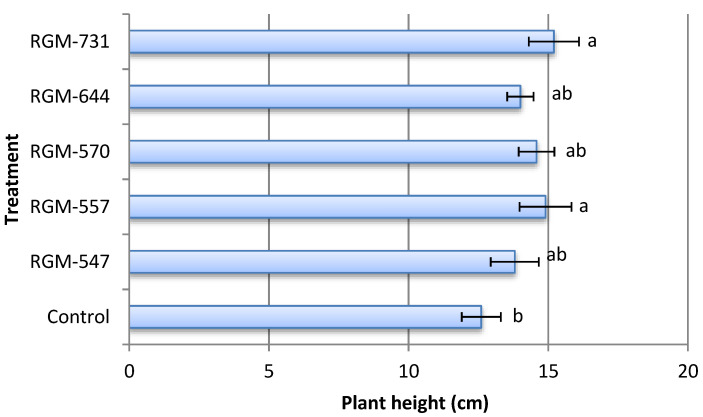
Effect of endophytic strains of *B. bassiana* on the mean height (±SE) of tomato plants (*Solanum lycopersicum*) 30 days after inoculation (1 × 10^6^ conidia mL^−1^). Different letters over the bars represent significant differences among the treatments according to Fisher’s LSD test (*p* < 0.05).

The inoculation of tomato seeds with native strains of endophytic *B. bassiana* had significant effects on the growth parameters studied. Thirty days after inoculation with strains RGM-557 and RGM-731 tomato plants presented a total height more than 21% and 18%, respectively, in comparison with the control that reached a height of 12.6 cm (F = 1.49; df = 5; *p* = 0.23). No significant differences were found among the treatments inoculated with diverse endophytic strains (Figure 6).

**Figure 7:**
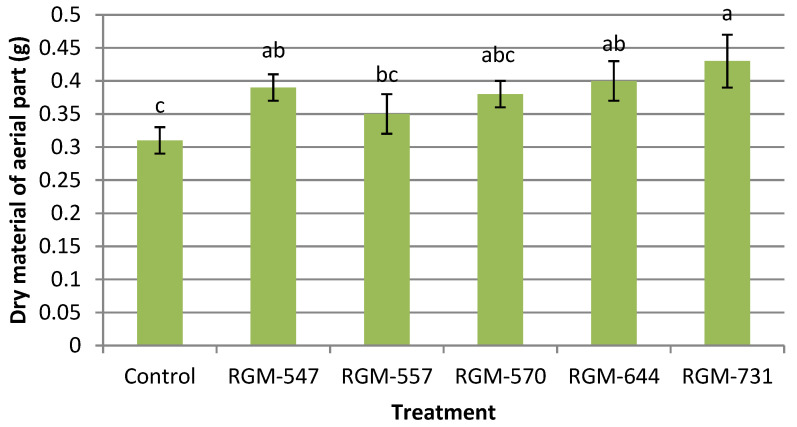
Effect of the endophytic strains of *B. bassiana* on the dry material of the aerial part (g) (±SE) of tomato plants (*Solanum lycopersicum*), 30 days after inoculation (1 × 10^6^ conidia mL^−1^). Different letters over the bars represent significant differences among the treatments according to Fisher’s LSD test (*p* < 0.05).

For the plant biomass production parameter that included the dry material from both the aerial (leaves and stems) and underground (roots) parts, significant differences were also found between the plants obtained from seeds treated with endophytes and the control plants. The aerial dry biomass reached significantly higher mean values when seeds were treated with *B. bassiana* endophytes in comparison to the control (F = 2.58; df = 5; *p* = 0.052). Plants from treated seeds with the strains RGM-547, RGM-644 and RGM-731 reached the highest aerial dry weight, while no significant differences were found among strains (*p* > 0.05), (Figure 7).

**Figure 8:**
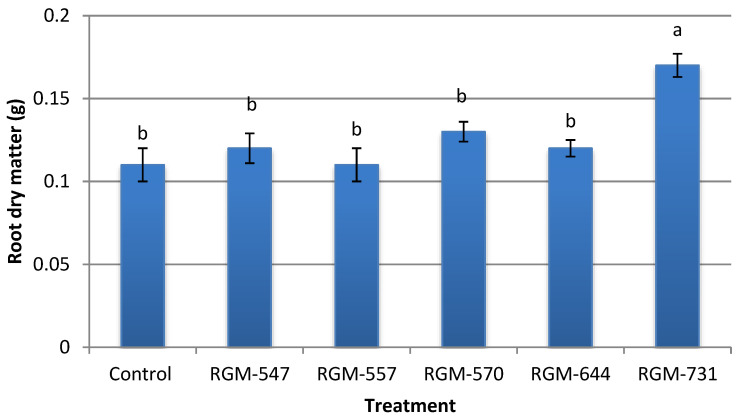
Effect of endophytic strains of *B. bassiana* on root dry matter (g) (±SE) of tomato plants (*Solanum lycopersicum*), 30 days after inoculation (1 × 10^6^ conidia mL^−1^). Different letters over the bars represent significant differences among the treatments according to Fisher’s LSD test (*p* < 0.05).

In the case of root dry weight, the mean weight fluctuated from 0.11 to 0.17 g. Plants from treated seeds with the strain RGM-731 reached a mean weight 55% higher than that of the control, (F = 5.2; df = 5; *p* = 0.002), while plants from treated seeds with the other four of *B. bassiana* strains presented no significant differences compared to the control (Figure 8).

## 4. Discussion

The five evaluated strains presented evidence of endophytic colonization in tomato leaves, which persisted until the end of the trial (Day 45). These results thus provide evidence of the growth-promoting activity of the native strains of endophytic *B. bassiana* in addition to their biocontrol action, as they were able to decrease the numbers of GWF eggs and nymphs on tomato plants.

### 4.1. Endophyte Effect on T. vaporariorum

Previous studies have provided evidence regarding the direct (entomopathogenic) effect of *B. bassiana* against GWF in tomato. It has been shown that strains of this fungus (Naturallis, ATCC_74040_, AL_1_, ALB_55_ y OF_13_) are pathogens to GWF, with a nymph mortality above 85% [12]. Meanwhile, Quesada-Moraga et al. [10] evaluated the effect of various strains of entomopathogenic fungi on nymphs of GWF in *Cucumis melo*, reaching mortality values over 50%. Although previous evidence has shown that *B. bassiana* can control GWF, this research is novel because it provides further details regarding the effects this fungus has as an endophyte against this pest [10,12]. Here, we demonstrated that the endophytic strains achieved a significant decrease in the number of eggs and nymphs in comparison to the uninoculated control, with strains RGM-557 and RGM-644 showing similar effects to the insecticide in relation to the decreased number of GWF eggs and strains RGM-557, RGM-644 and RGM-731 showing a significantly higher reduction in the number of nymphs compared to the synthetic insecticide.

Powell et al. [45] demonstrated a reduction in the survival of *Helicoverpa zea* larva when fed on tomato plants inoculated with strains of endophytic *B. bassiana*. The population reduction of *H. zea* as a consequence of the endophytic action of *B. bassiana* and *Purpureocillium lilacinum*, was also reported by Lopez and Sword [46] in cotton (*Gossypium hirsutum*). On the other hand, it has been shown [47] that *Vicia faba* L. seeds treated with endophytic fungi induced systemic changes in the plant, negatively affecting the behavior of aphids *Acyrthosiphon pisum* and *Aphis fabae*. Although a significant number of studies have reported the antagonist effects of endophytic fungi against various insects, the mechanisms involved in their action have not yet been clearly demonstrated and reported.

The decrease in the number of eggs on plants inoculated with endophytic strains in our study could be explained by GWF adults preferring to lay their eggs on uninoculated plants instead of inoculated plants [48]. On the other hand, this study showed that the GWF is most susceptible to the negative effects of the evaluated endophytic strains in its nymph stage. Studies by Mascarin et al. [49], have also shown that entomopathogenic fungi are more effective in controlling GWF in their nymph stage; they related this effect with the direct contact between the nymphs and fungi conidia in foliar applications, considering their limited mobility. Nevertheless, in our study, the epiphytic action of the fungus was discarded because the conidia of the fungus did not come into direct contact with the insect when were applied (substrate inoculation). Furthermore, there was no evidence of *B. bassiana* growth in GWF adults under incubation, suggesting that the decrease in nymph population could be attributed to the presence of the fungus within the plant. In a review of several studies by Vega [14], where entomopathogenic fungi were used for insect control, 93% of these provided evidence of the absence of mycosis in insects, demonstrating its effect as an endophyte. Studies by Menjivar et al. [48] showed a decrease in GWF in tomato plants (50 to 70% less insects) when roots were inoculated with the endophytic fungus from the genus *Fusarium*. Several authors have affirmed that the negative effect in the early stages of insect development due to entomopathogenic fungal endophytes, as in our study, are related to the production of toxic substances (secondary metabolites) in the plant tissues [50,51,52,53] and/or due to the changes in bioactive compounds induced in plants by the endophytic fungus [47].

Studies by Xu et al. [54,55] with *B. bassiana* have demonstrated that bassianolide and beauvericin metabolites function as virulence factors against various insects. It has also been reported that *Beauveria* produces the bassiacridin metabolite which has a toxic action in insects [56]. The above suggests that the production of compounds in the plants also could inhibit the insect from searching for plants [57]. On the other hand, the decrease in the number of eggs and nymphs could also be related to a plant response against insects mediated by endophytic fungi, resulting in the production of secondary metabolites with toxic, repellent or anti-feeding effects for insects [14,48,58]. It is possible that the endophytic fungi negatively affect the insect population as a result of the plant induced systemic response [59], which can occur far from where the elicitor was inoculated [60].

According to the manufacturer’s recommendations, INSEGAR^®^ 25 WG should be applied at the beginning of the tomato crop and repeat its application three times. If a 130-day tomato crop cycle is considered, this product could provide protection for periods of 40 days. In the first period of time, the endophytic strains RGM-557 and RGM-644 presented better results than this insecticide in reducing the number of eggs and nymphs. The endophytic fungi could complement or substitute the use of this chemical insecticide in the control of whiteflies.

Our study provided evidence of the negative effect of the endophytic fungi strains on the number of GWF eggs and nymphs on tomato leaves. Nevertheless, future studies must further examine the mechanisms that cause these responses.

### 4.2. Growth Promotion

Our results showed that the endophytic strains used in this study exerted a growth promotion effect, which led to taller plants and greater aerial and root dry weights, in most cases superior to the uninoculated control. This confirms the protocooperation between the plant and the endophytic fungus, which improved the growth of the plant. Various studies have confirmed the positive effects of strains of entomopathogenic fungal endophytes in the growth of different crops such as tomato, wheat, corn, cotton and bean [17,20,27,32,46,61]. Our results coincide with those presented by Sánchez-Rodríguez et al. [62], where they demonstrated the ability of *B. bassiana* to internally colonize tomato plants without negatively affecting plant height and biomass production. Tall and Meyling [27] also provided evidence of the growth promoting effect of the endophytic strains of *B. bassiana*, which when applied to *Zea mays* seeds had a positive effect on plant growth in a substrate with a high nutrient content. Studies by Sánchez-Rodríguez et al. [35] in *Triticum aestivum* demonstrated that seed inoculation with the endophytic *Beauveria* showed no significant differences in comparison to the uninoculated control in terms of plant height during the first 17 days after inoculation, which could explain the energy cost the plant must pay in order to tolerate the endophyte. Nevertheless, 23 to 31 days after the inoculation they found significant differences in height. A significant increase in the dry weight of the spikes was also registered. It is important to mention that, similar to a study by García et al. [20] with *M. anisopliae* endophytes, our study observed that the growth promoting effect is dependent upon the fungal strain, as significant differences were observed among strains. In our study, the strain RGM-731 showed the most promising results in terms of plant height and root and shoot dry matter.

In relation to the mechanisms associated with plant growth promotion activity by endophytic fungi, we first ruled out the growth promotion effect through indirect mechanisms, since the plants were not affected by pests or diseases during the completion of the assays. Therefore, the growth promoting activity could be explained by direct mechanisms such as the production of phytohormones or growth regulators (metabolites)—in addition to the bioavailability of the necessary nutrients for the growth of plants. In this sense, this study discards, in part, the effect associated with the production of phytohormones, specifically indole compounds such as indoleacetic acid, because preliminary studies of this strain were unable to produce them. Our results could be more related to the increase in the bioavailability of nutrients and could be explained in part by the production of iron siderophores and phosphate solubilization. It cannot be ruled out that other types of hormones could have been produced, such as cytokinins or gibberellins, which have been widely reported in association with bacteria and some fungi linked to plants as endophytes [63,64,65].

In the present study, only the endophytic strain RGM-547 showed no ability to solubilize phosphate, while the other four strains were able to form solubilization halos, which, according to Perez et al. [66], could indicate different degrees of efficiency of phosphate solubilization. In this case, the strains RGM-731 and RGM-557 reached the highest efficiency. Studies by Pal and Ghosh [67] have also provided evidence that *B. bassiana* has the ability to solubilize phosphate. The strain used in their study produced phosphate solubilization halos of 1.4 cm at Day 14, while the endophytic strain RGM-644, used in the present study, reached halos of 1.24 cm; though, these measurements were taken after only 10 days. The endophytic strains that solubilize phosphate could provide an important quantity of available phosphate for plant growth, which could explain the growth promoting activity induced by the endophytic strains found in the present study in tomato.

On the other hand, the ability to produce iron siderophores by entomopathogenic fungi has been reported by various authors and in all cases it has been related with siderophores of the hydroxamate type [68,69,70]. The present study confirmed that the evaluated native strains of entomopathogenic endophytes can indeed produce siderophores, which could be associated with the ability that these fungi must produce secondary metabolites of diverse types. Studies by Jirakkakul et al. [31] relate the siderophore production of *Beauveria* with tenellin, a metabolite produced by this fungus that could act as an iron chelator, associated with the accumulation of ferricrocin in the fungus hyphae. The significant siderophore production by the strain RGM-731 could leave iron available for the plant, which could lead to a higher dry biomass. Studies by Sánchez-Rodríguez et al. [62] in tomato plants demonstrated that the endophytic *B. bassiana* is capable of increasing the bioavailability of iron in calcareous substrates, which stimulated both shoot and root growth.

Our results, in terms of the growth promotion and decrease in the GWF population in tomato plants, is concurrent with that presented by Jaber and Ownley [71] in the sense that the entomopathogenic fungi can live as endophytes and contribute to insect pest suppression, as well as to plant growth promotion.

## 5. Conclusions

The results obtained in this study suggest that tomato seed inoculation with endophytes could be used as a growth-promoting alternative. Furthermore, the evaluated strains show potential for GWF biologic control and could replace or be used in conjunction with insecticides, with consequent environmental, social and economic benefits. However, it is necessary to validate these results in the field since endophyte behavior may be modified by uncontrolled environmental variables. This is also necessary to deepen our understanding of the mechanisms involved in the effect that endophytes have on GWF. On the other hand, it is necessary to explore the results that could be obtained by combining the action of *B. bassiana* as an epiphyte and as an endophyte against the GWF, since its antagonist action could be enhanced by other control mechanisms.

## Figures and Tables

**Table 1:** Fungal strains assessed in this study.

Code Strain *	Species	Origin	Habitat
RGM-547	*Beauveria bassiana*	Santa Bárbara, Biobío Region, Chile.	Natural pasture soil
RGM-557	*Beauveria bassiana*	Los Lagos, Los Lagos Region, Chile.	Natural pasture soil
RGM-570	*Beauveria bassiana*	Molina, Maule Region, Chile.	*Vitis vinifera*, vineyard soil
RGM-644	*Beauveria bassiana*	Icalma, La Araucanía Region, Chile.	Natural pasture soil
RGM-731	*Beauveria bassiana*	Río Cisnes, Aysén del General Carlos Ibáñez del Campo Region, Chile.	Natural pasture soil

* Accession number of microorganisms from the Chilean Collection of Microbial Genetic Resources—CChRGM.
